# Supplementary material for: Learning to detect AI texts and learning the limits
Source: PLoS One. 2025 Oct 15;20(10):e0333007. doi: 10.1371/journal.pone.0333007 (PMC12527182; doi:10.1371/journal.pone.0333007)
Supplement: S2 File — All data and scripts used in the study, some additional descriptive statistics, scalable formate figures. (ZIP) [file pone.0333007.s002.tgz › data and scripts/OSF/scripts/analysis/Dependencies/GLS2_Assistant_vs_Confidence.pdf]

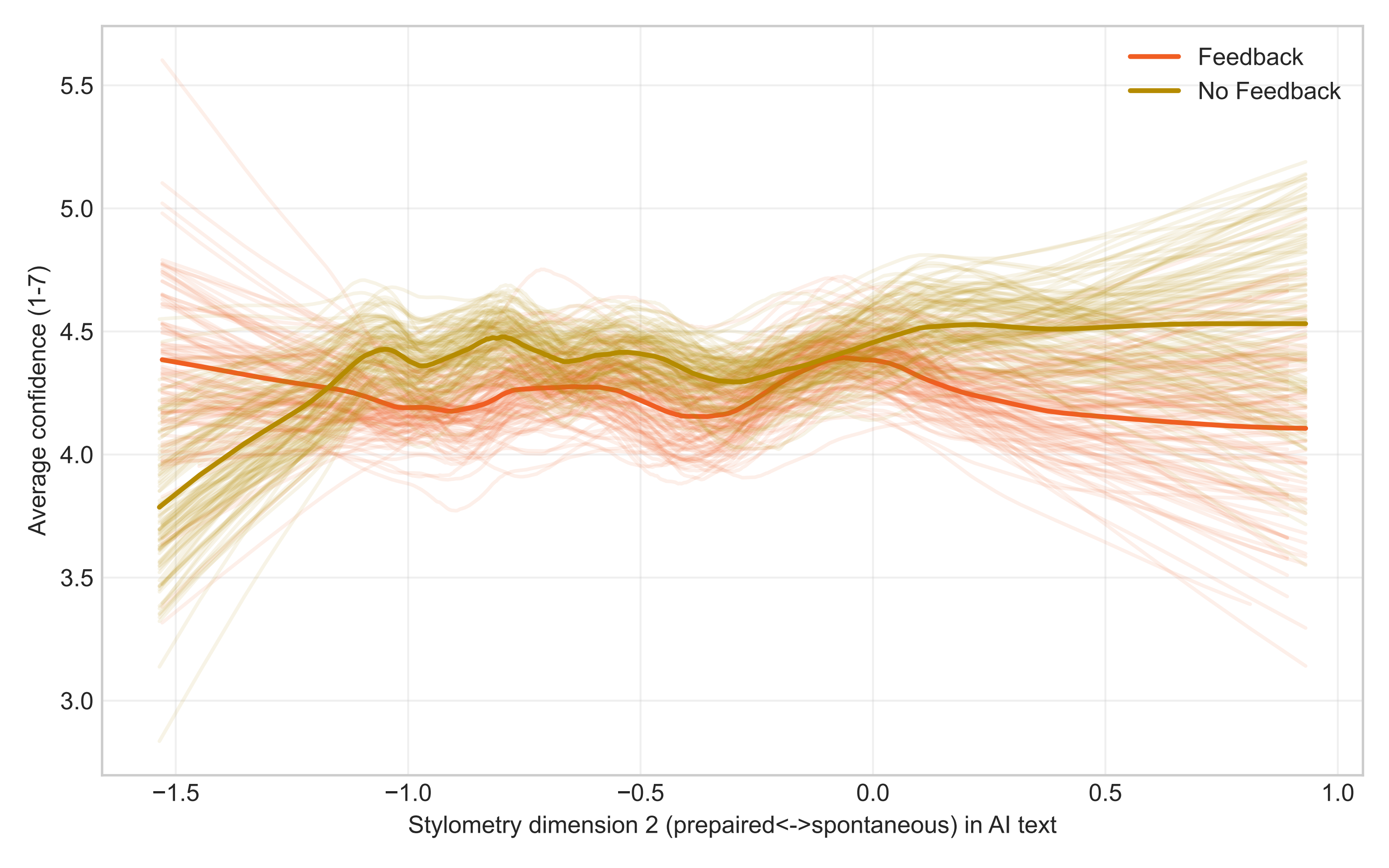

Feedback
No Feedback
Average confidence (1-7)
5.5
5.0
4.5
4.0
3.5
3.0
−1.5
−1.0
−0.5
0.0
0.5
1.0
Stylometry dimension 2 (prepaired<->spontaneous) in AI text
